# Structural Insights into Mouse H-FABP

**DOI:** 10.3390/life12091445

**Published:** 2022-09-16

**Authors:** Lili Wang, Haoran Zhang, Panjing Lv, Yan Li, Maikun Teng, Yahui Liu, Donghai Wu

**Affiliations:** 1 School of Life Science, University of Science and Technology of China, Hefei 230027, China; 2 Key Laboratory of Regenerative Biology, Guangdong Provincial Key Laboratory of Stem Cell and Regenerative Medicine, Guangzhou Institutes of Biomedicine and Health, Chinese Academy of Sciences, Guangzhou 510530, China; 3 Department of Pathogen Biology, School of Basic Medicine, Tongji Medical College, Huazhong University of Science and Technology, 13 Hangkong Road, Wuhan 430030, China; 4 China-New Zealand Joint Laboratory on Biomedicine and Health, Guangzhou 510530, China

**Keywords:** mouse H-FABP, crystal structure, NMR structure, structural biology

## Abstract

Intracellular fatty acid-binding proteins are evolutionarily highly conserved proteins. The major functions and responsibilities of this family are the regulation of FA uptake and intracellular transport. The structure of the H-FABP ortholog from mouse (*Mus musculus*) had not been revealed at the time this study was completed. Thus, further exploration of the structural properties of mouse H-FABP is expected to extend our knowledge of the model animal’s molecular mechanism of H-FABP function. Here, we report the high-resolution crystal structure and the NMR characterization of mouse H-FABP. Our work discloses the unique structural features of mouse H-FABP, offering a structural basis for the further development of small-molecule inhibitors for H-FABP.

## 1. Introduction

The intracellular fatty acid-binding proteins (FABPs), a family of 14–15 kDa proteins, are divided into at least nine distinct types, named according to the organ where they were first identified or the expression predominance. Examples include heart-type FABP (H-FABP), intestinal-type FABP (I-FABP), liver-type FABP (L-FABP) and adipocyte-type FABP (A-FABP, also known as aP2 and FABP4) [1,2,3,4]. H-FABP is mainly expressed in cardiomyocytes, skeletal muscle, and several other tissues such as the brain, adrenal gland, kidney, mammary gland, and blastocysts [5,6]. It is highly conserved in evolution, and its primary role is the regulation of fatty acid (FA) uptake and intracellular transport [7,8,9].

Known FABP members are composed of 10 anti-parallel beta strands that form a β-barrel structure capped by a short helix-turn-helix motif [10]. FABPs are capable of binding a variety of fatty acids in the barrel cavity and are involved in FA uptake, long-chain FA transport, and cellular FA metabolism [11]. In cardiomyocytes, H-FABP binds to long-chain FAs and transports them from the cytoplasmic membrane to the sites of lipidation and hydrogenation, where FAs are eventually oxidized to produce ATP and provide energy for myocardial contraction [12,13]. H-FABP demonstrates a preferential binding to the n-6 FA family, suggesting a potential role in the trafficking of arachidonic acid (20:4) [14,15,16]. Anandamide, another FA which is linked to inflammation and pain, is transported by FABP5 and FABP7. Inhibitors designed for FABP5 and FAPB7 are candidates for the production of anti-inflammatory drugs [17,18]. Anandamide is known to participate in many crucial physiological processes; in particular, it is postulated to modulate the intracellular concentration of FAs and influence the function of enzymes, ion channels, and receptors [15].

Because of its high sensitivity and specificity, the H-FABP gene can be used to diagnose and evaluate the severity of heart failure in different types of heart disease, and can also be a practical biomarker of myocardial injury [19]. H-FABP is abundantly present in the myocellular cytoplasm, with rare content in the plasma and urine of healthy individuals. H-FABP is rapidly (<1 h) released into the plasma after the onset of myocardial injury, resulting in a sharp increase in serum concentrations of H-FABP [19]. It has been clinically confirmed that H-FABP concentration can estimate the area of myocardial damage [20]. Thus, H-FABP is becoming an effective biomarker for the diagnosis and assessment of cardiovascular diseases, such as congestive heart failure (CHF), unstable heart pectoris (UAP), acute myocardial infarction (AMI), dilated cardiomyopathy (DCM), and many other diseases [19]. It can also be used as a biomarker for detecting ischemic injury in donor kidney perfusates [21]. In addition, H-FABP, known as a mammary-derived growth inhibitor (MDGI), is suggested to be associated with tumor proliferation, and is shown to have a preliminary role in inhibiting tumor growth [22]. It has also been demonstrated that H-FABP regulates skeletal muscle aging and is considered a therapeutic target for intervention in sarcopenia, and is used as a biomarker for the diagnosis of skeletal muscle necrosis in rats [23,24]. Recently, H-FABP has been proposed as a non-specific disease-related biomarker of neurodegeneration due to its elevated levels in cerebrospinal fluid (CSF) [25]. The significant role H-FABP plays in clinical treatment and diagnosis renders it a valuable biotarget for investigation.

Currently, the three-dimensional structures of H-FABP in humans and bovines are determined by X-ray or NMR techniques [26,27,28]. The structure of the H-FABP ortholog from mouse (*Mus musculus*), one of the most important model organisms for animal experiments, had not been revealed at the time this study was completed. Thus, further exploration of the structural properties of mouse H-FABP is expected to extend our knowledge of the model animal’s molecular mechanism of H-FABP function. In order to further investigate the structural basis of H-FABP in mouse, we report here the expression, purification, crystal structure and NMR characterization of the recombinant mouse H-FABP.

## 2. Materials and Methods

### 2.1. Plasmid Construction

The full-length cDNA for mouse H-FABP was amplified using the polymerase chain reaction (PCR) method from mouse (C57BL/6) heart cDNAs, using the forward primer F (TTTGGATCCATGGCGGACGCCTTTG) and the reverse primer R (GTTGTCGACTCACGCCTCCTTCTC). The PCR product was then digested by restriction enzymes Sal I and BamH I, after which the digestion product was purified and ligated to pET28a bacterial expression plasmids cut by the same restriction enzyme pair. The ligation products were transformed into *E. coli* Top10 competent cells, and successful cloning was confirmed by PCR and DNA sequencing.

### 2.2. Protein Expression and Purification

*E. coli* BL21(DE3) bearing the recombinant expression plasmid H-FABP-pET28a was cultured overnight, inoculated into a 1 L LB medium containing 50 μg/mL kanamycin, and grown at 37 °C to an OD_600_ of 0.6. Subsequently, the cultures were cooled to 16 °C, and 0.1 mM IPTG was added to induce mouse H-FABP protein expression for 22 h. 

To obtain the ^15^N/^13^C-labeled mouse H-FABP protein, after the overnight incubation at 37 °C in 1 L LB medium, the bacterial cells were pelleted by centrifugation and transferred into a 500 mL of M9 minimal medium (containing 1 g/L of NH_4_Cl (^15^N, 99%) and 2 g/L of D-glucose (U-^13^C_6_, 99%) as the sole nitrogen and carbon sources, respectively) to obtain a starting OD_600_ of 1.0–1.5. The cell cultures were incubated at 37 °C for 1 h and then 0.1 mM IPTG was added to induce the ^15^N/^13^C-labeled mouse H-FABP protein at 16 °C for 22 h.

After being harvested by centrifugation, cells were resuspended and lysed by pulsed sonication in buffer A containing 20 mM Tris-HCl and 500 mM NaCl, 5% Glycerol, pH 7.8. The supernatant of the lysate was loaded onto a Ni-chelating sepharose fast flow (GE Healthcare, The Bronx, NY, USA) column, pre-equilibrated with buffer A. Unbound and non-specifically bound proteins were washed down with buffer A containing 20 mM imidazole. Eventually, the H-FABP proteins fused with a histidine_6_-tag (HexHis) at the N-terminal were eluted in buffer A containing 200 mM imidazole. The proteins were concentrated by ultrafiltration in a Millipore Amicron Ultra ultrafiltration device (3 kDa cutoff). The protein HexHis-H-FABP was further purified by gel filtration chromatography Superdex 75 (GE Healthcare, The Bronx, NY, USA) in buffer B containing 20 mM Tris-HCl, 200 mM NaCl pH 7.8. The purity of the proteins was estimated by Tricine SDS-PAGE. 

### 2.3. Crystallization

The protein was desalted by ultrafiltration (Millipore, 3 kDa cutoff) until the concentration of NaCl was below 50 mM. The concentration of purified protein was determined by the BCA protein assay with BSA as the standard. The final protein concentration was about 100 mg/mL. An amount of 97 mg/mL of the recombinant mouse H-FABP in 20 mM Tris-HCl, 50 mM NaCl pH 7.8 was used for the initial screen of the crystal growth condition. The crystals were grown using the hanging-drop vapor-diffusion method at 14 °C. The initial screen of the crystal growth condition was performed with the Hampton crystal screen kit and Hampton crystal screen II kit. Each hanging drop consisted of 1 µL reservoir solution and 1 µL protein solution, equilibrated against 100 µL reservoir solution. Small needle-like crystals appeared 4 days later and grew into a bigger size in about 10 days in 200 mM (NH_4_)_2_SO_4_, 100 mM CH_3_COONa, and 25% PEG 4000. After several rounds of optimization of protein concentration, precipitant concentration, crystallization temperature, buffer pH, and buffer concentration, crystals suitable for X-ray diffraction were obtained in two conditions: 25% PEG 2000 MME, 100 mM HEPES, pH 7.5, or in 25% PEG 4000, 100 mM sodium cacodylate, pH 6.5 using 100.5 mg/mL H-FABP at 14 °C grown in 4 days.

The initial X-ray diffraction experiments were performed using Cu K X-rays generated by a RA-Micro007 rotating-anode X-ray source (Rigaku, Tokyo, Japan) and the diffraction images were collected using a MAR 345dtb imaging-plate detector in the lab of USTC. The crystals were quickly passed through a cryoprotectant buffer containing 20% glycerol, 25%PEG 2000 MME, 0.1 M HEPES pH7.5 and flash-cooled in a stream of cold nitrogen gas. A complete diffraction data set consisting of 182 images was collected with an oscillation angle of 1^o^ per image at 100K in home source. 

The diffraction data were processed and scaled using iMosflm and programs from CCP4 (Collaborative Computational Project, Number 4, 1994).

### 2.4. Structure Determination and Comparison

The X-ray diffraction datasets for FABP were collected in the USTC lab, and all diffraction data were indexed, integrated, and scaled using HKL2000 [29]. The structure was solved with molecular replacement with the model of Human H-FABP (PDB ID: 1HMR). The initial model was built and fitted with Coot [30]. Refinement was performed for several cycles with PHENIX [31]. All figures were drawn using PyMOL [32]. The crystallographic and refinement statistics are listed in Table 1.

### 2.5. NMR Experiments and Data Processing

The [^15^N]-labeled and [^15^N,^13^C]-labeled mouse H-FABP proteins were further purified by gel-filtration chromatography Hiload 16/600 Superdex 75 pg (GE Healthcare) in a buffer containing 20 mM HEPES, 150 mM NaCl pH 7.0. The proteins were concentrated to about 0.7 mM by ultrafiltration (Millipore, 3 kDa cutoff).

Samples of 0.7 mM [^15^N]-labeled (for 2D NMR experiments) or [^13^C, ^15^N]-labeled (for 3D NMR experiments) mouse H-FABP proteins in the NMR buffer (20 mM HEPES, 150 mM NaCl, pH 7.0) were prepared for NMR spectroscopy. For backbone resonance assignment, 2D ^1^H-^15^N HSQC and 3D CBCANH, CBCA(CO)NH, HNCA, HN(CO)CA, HNCO, HN(CA)CO spectra were acquired. The above NMR experiments were conducted via a Bruker Avance 600 MHz spectrometer equipped with a triple-resonance cryogenic probe at 298 K.

A sample of 1.0 mM [^15^N]-labeled FABP proteins in the NMR buffer was used to conduct ^15^N relaxation measurements, including T_1_ and T_2_ relaxation times and the ^1^H-^15^N steady-state nuclear Overhauser effect (hNOEs) of backbone amide groups. The above relaxation parameters were measured on the Bruker Avance 600 MHz spectrometer at 298 K. T_1_ measurements are based on 2D ^1^H-^15^N correlation data collected with relaxation delays of 11.2, 61.6, 142, 243, 364, 525, 757 and 1150 ms, and duplicated data at the fourth time point were acquired to estimate errors. For T_2_ measurements, delays of 0, 17.6, 35.2, 52.8, 70.4, 105.6, and 140.8 ms were used, and the data at the fourth time point were repeatedly recorded for error estimation. The hNOEs were calculated using pairs of ^1^H-^15^N correlation data acquired with and without amide ^1^H saturation.

Maximum peak intensities were used to process R_1_, R_2_ and hNOE data. Peak intensity decays were fit to mono-exponential functions, and the standard deviations were estimated with 10,000 times of fitting for the Gaussian distributed random error of peak heights. The NMR data were processed and analyzed with NMRPipe [33] and POKY [34].

The rotational correlation time *τ_c_* of H-FABP was calculated using R_2_/R_1_ ratios under the protocol proposed by Kawale et al [35]. The empirical formula for the residue-wise *τ_c_* calculation was:

$$\tau_{c}\approx \frac{1}{4\pi \nu_{N}}\sqrt{6\frac{T_{1}}{T_{2}}-7}$$

where *ν_N_* is the resonance frequency of the ^15^N nuclei (60.84 × 10^6^ Hz in this study). 

The molecular weight *M_w_* was estimated with the following empirical equation, which was a least-square fit to the *M_w_-τ_c_* data of a serial of typical globulins [36] (R^2^ = 0.9284):

$$M_{W}\approx 1.4936\tau_{c}+1.1187$$

where *M_W_* is given in kDa and *τ_c_* in ns.

## 3. Results and Discussion

### 3.1. Crystal Structure of Mouse H-FABP

**Figure 1:**
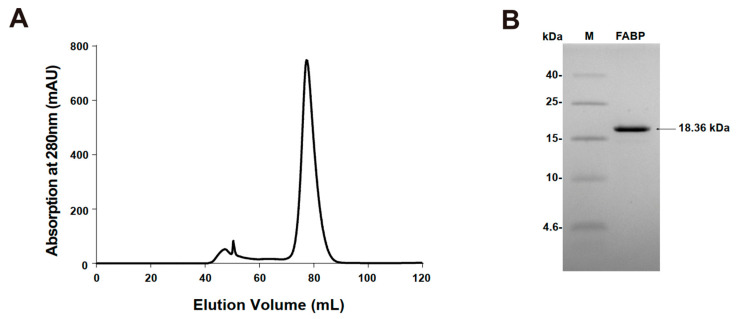
Purification of FABP. (**A**) FABP were separated using HiLoad 16/600 Superdex 75 gel filtration chromatography. Tricine SDS-PAGE of purified FABP. Lane M: protein molecular marker (low-range protein ladder); Lane FABP: purified protein FABP. (**B**) SDS-PAGE of purified FABP.

**Figure 2:**
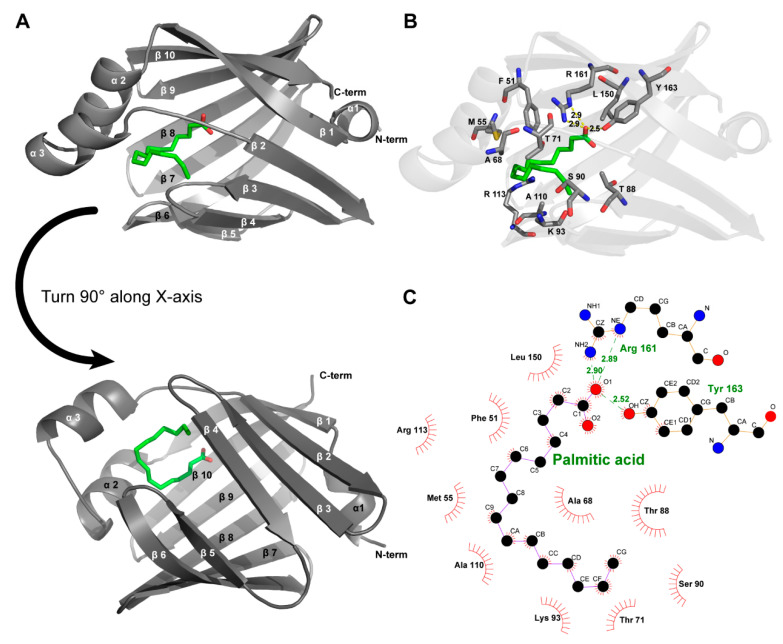
Crystal structure of mouse H-FABP. (**A**) General overview of the crystal structure of the complexes of FABP with palmitic acid (PDB entry ID:7YF1); (**B**) amino acids interact with palmitic acid on FABP proteins, including hydrophobic interactions and hydrogen bonds (yellow dashed lines). (**C**) Two-dimensional interactions demonstrated via Ligplot+.

Mouse H-FABP, a fatty acid-binding protein from *Mus musculus*, has 133 a.a. (amino acid) residues, a theoretical pI (isoelectric point) of 6.11, and a calculated *M_w_* (molecular weight) of 14.82 kDa. Mouse H-FABP was successfully expressed in *E. coli* and highly purified to homogeneity with an N-terminal 6 His tag (167 a.a., a pI of 8.04 and an *M_w_* of 18.36 kDa for the recombinant H-FABP) (Figure 1A,B). The crystal of mouse H-FABP belongs to space group P2_1_2_1_2_1_ at a resolution of 1.5 Å. The protein was crystallized from 25% PEG 2000 MME, 0.1 M HEPES pH 7.5 and 25% PEG 4000, 0.1 M sodium cacodylate pH 6.5. Both the Matthews coefficient analysis and the self-rotation function suggest the presence of one molecule per asymmetric unit in the mouse H-FABP crystal. One ligand molecule, the palmitic acid, was determined to be present inside the barrel cavity of H-FABP per asymmetric unit. The FABP–palmitic acid complex was formed during the expression of the FABP protein in *E. coli*. H-FABP adopted a β-barrel-like conformation, composed of 3 α-helices (α1: 35–39, α2: 51–58, α3: 62–68) and 10 anti-parallel β-strands (β1: 41–49, β2: 74–80, β3: 83–89, β4: 94–99, β5: 105–109, β6: 112–122, β7: 125–132, β8: 135–144, β9: 147–154, β10: 157–165). Notably, the lack of backbone hydrogen bonds between β4 and β5 prevented the two strands from forming a typical anti-parallel β-sheet (Figure 2A). Instead, hydrophobic interactions were observed among the nonpolar residues of β4 (I97 and F99) and β5 (F105), conceivably stabilizing the β-barrel and the ligand’s nonpolar tail (Figure 2B,C). The Arg161 and Tyr163, which localized in β10, had direct interaction with the carboxylate group of FA. The tail of FA was inserted into the hydrophobic core, which was formed with Leu150 in β9, Phe51, Met55 in α2, Ala68 in α3, and Ala110 between β5 and β6 (Figure 2B,C). Although the human H-FABP crystal structure has been solved for several years (PDB entry ID: 1HMR), structural discrepancies between the H-FABP structures in human and mouse were evident. Comparatively, mouse H-FABP has one more α-helix (α1) around the helix-turn-helix structure. We suppose that the (α1) might be helpful for a tighter interaction with a different binding partner. The performance of further biochemical and structural analysis may be required to fully understand the functional significance of the structural differences between human and mouse H-FABP.

### 3.2. NMR Assignment and Secondary Structure Prediction of Mouse H-FABP

**Figure 3:**
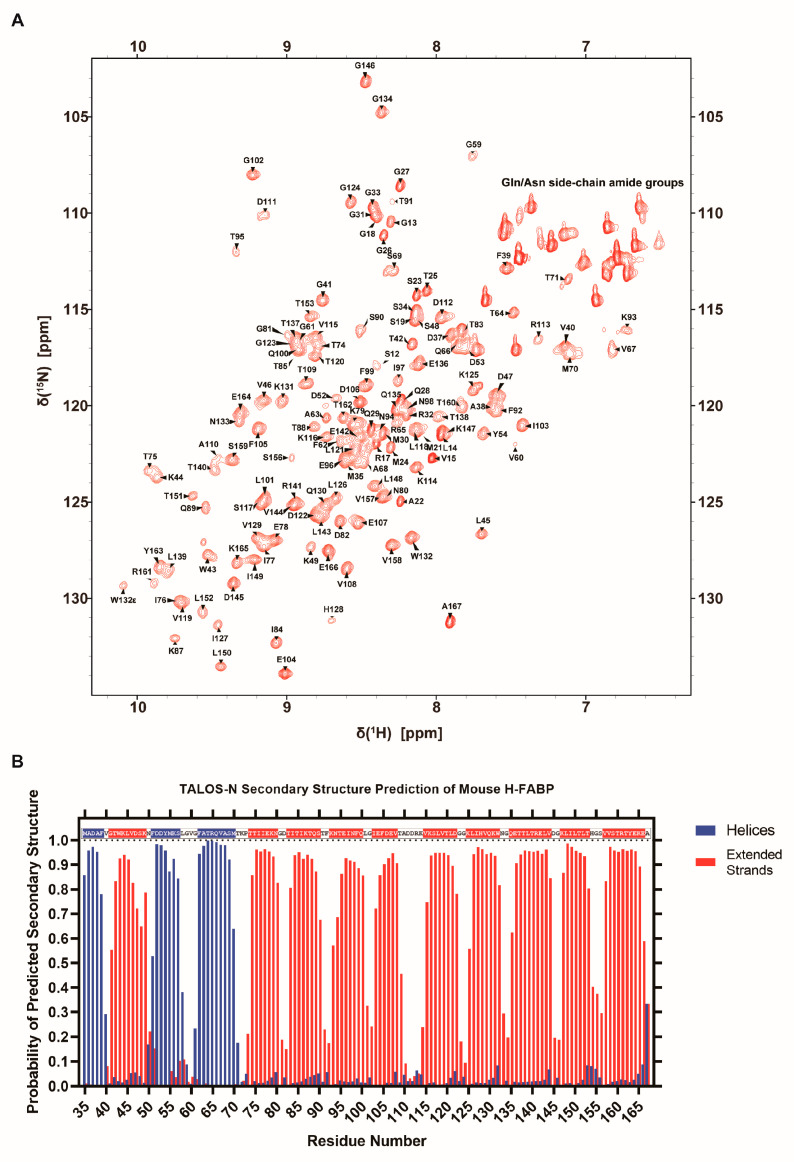
NMR characterization of H-FABP. (**A**) ^1^H-^15^N HSQC spectrum of H-FABP with resonance assignment indication of individual peaks. (**B**) Column graph illustrating the predicted probability of H-FABP secondary structure via TALOS-N [37]. Residues predicted to have over a 50% probability of adopting a helical or a stranded secondary structure are indicated within the one-letter sequence on the inner upside of the graph in the same color scheme.

To initiate the NMR-based characterization of mouse H-FABP, we assigned the chemical shifts of H-FABP backbone atoms. In total, 92.4% of the backbone amide ^1^H-^15^N pairs were assigned for the fusion-tag-excluded H-FABP (a.a. 35–167, hereafter referred to as H-FABP_35–167_) (Figure 3A). Backbone amide groups of residues A36, N50, F51, M55, K56, S57, L58, I86, H154, and G155 were not assigned due to the affirmed signal absence (for example, the absence of L58 C_α_ signal in the G59 C_α_-related strips) or the consequently unsuccessful serial connection. Assignment completeness was 95.5% for C_α_, 72.4% for C_β_, and 88.7% for C(O). These assignment data were deposited into the Biological Magnetic Resonance Bank (BMRB) under accession number 51545.

The chemical shift list was subsequently passed to the TALOS-N server [37] for secondary structure prediction. As a result, H-FABP_35–167_ was predicted to consist of 3 helices (α1: 35–39, α2: 51–57, α3: 62–70) and 10 β-strands (β1: 41–49, β2: 74–80, β3: 83–90, β4: 93–100, β5: 103–108, β6: 115–122, β7: 125–132, β8: 135–144, β9: 147–153, β10: 157–166) (Figure 3B). This result was comparable to our aforementioned experimental result of H-FABP (Figure 2A). As TALOS-N relies on the hybridization of secondary-chemical-shift-based and sequence-based prediction methodologies [37], the similarity of identification of secondary structure elements implies a high structural consistency of H-FABP in an NMR solution and in a crystallization environment.

### 3.3. NMR Dynamics of Mouse H-FABP

With the overall backbone assignments of mouse H-FABP_35–167_, we further performed an NMR dynamics analysis on the protein. Longitudinal relaxation rate *R*_1_, transverse relaxation rate *R*_2_, and ^1^H-^15^N steady-state nuclear Overhauser effect (hNOE) are the parameters for characterizing protein dynamics. ^15^N relaxation experiments were conducted to collect data on *R*_1_, *R*_2_, and hNOE for each residue. A total of 111 non-proline residues were analyzed, with residues suffering from overlapped peaks or low signal-noise ratio excluded.

**Figure 4:**
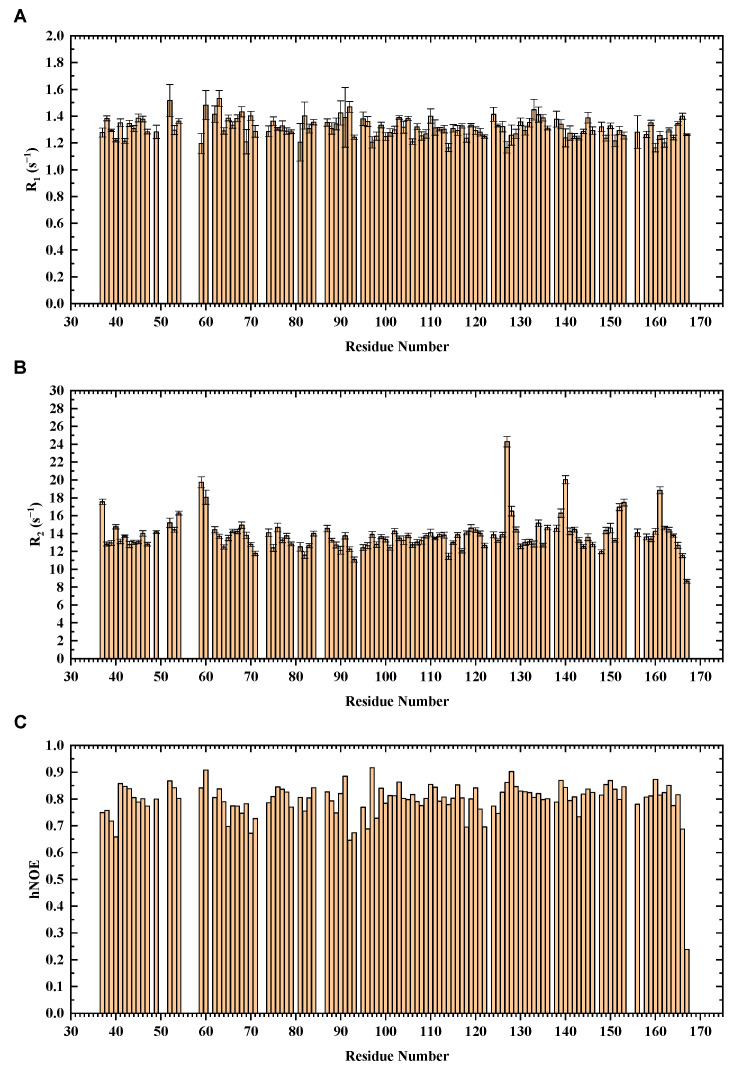
NMR ^15^N relaxation analysis of H-FABP. The relaxation parameters *R*_1_ (**A**), *R*_2_ (**B**), and hNOE (**C**) are indicated in column graphs.

The residual relaxation parameters’ distribution exhibited a concentrated distribution (Figure 4). The global average of hNOE for H-FABP_35–167_ reached 0.80, indicating a compact β-barrel capped with α-helices. Residual hNOEs and secondary structure elements share a similar pattern of residue-wise fluctuation. Interestingly, the apparent *R*_2_ values of G59, V60, I127, T140, L152, T153, and R161 were significantly higher than their neighboring residues, implying the existence of the conformational change rates R_ex_ as a linear term of *R*_2_ values. Inspection of the crystal structure of H-FABP revealed that G59, V60, L152 and R161 were in direct contact with the palmitic acid through hydrophobic interactions or a potential salt bridge, suggesting a dynamical protein–ligand interaction pattern, should the R_ex_ values of these residues be confirmed. I127, T140 and T153 were spatially adjacent β-barrel residues with side chains stretching outside the barrel; the specific functions these three residues were engaged in were not explicit. Future investigation of the delicate dynamics pattern of H-FABP is expected to illustrate these unique dynamics properties further.

Relaxation data have also been widely used to estimate globulin’s molecular weight by determining the rotational correlation time, which is the time for a protein molecule to rotate by one radian and is thus a reflection of the protein’s molecular size [36]. The rotational correlation time *τ_c_* was calculated to be 10.82 ns using the *R*_2_/*R*_1_ ratios of H-FABP_35–167_, and the consequent estimation of *M_w_* of the H-FABP molecule was 17.28 kDa. Since the theoretical *M_w_* is 14.82 kDa for segment H-FABP_35–167_ and 18.36 kDa for the full-length recombinant H-FABP, the similar relaxation-derived *M_w_* suggested the monomeric status of H-FABP in solution.

## 4. Conclusions

In this study, we successfully prepared the protein sample of mouse H-FABP, resolved the crystal structure of mouse H-FABP, and performed an NMR-based characterization of H-FABP. Our results disclosed the unique structural properties of mouse H-FABP, laying a structural basis for the further development of small-molecule inhibitors for H-FABP. The development of highly active and specific H-FABP inhibitors has important theoretical research significance and clinical translational value. However, since H-FABP and A-FABP share highly similar amino acid sequences and three-dimensional structures, the selectivity of compounds for H-FABP and A-FABP must be considered during the development of FABP inhibitors. Thus, the determination of a high-resolution structure is critical for understanding the structural basis of H-FABP and A-FABP, and for discovering new selective FABP inhibitors. Future research should focus on both increasing the activity and selectivity of inhibitors and considering issues such as the synergistic effects of inhibitors on other FABP family members.

## Figures and Tables

**Table 1:** X-ray data collection and processing statistics. Values in parentheses are for the last shell.

Data Collection Statistics	
Wavelength (Å)	1.54178
Space group	P2_1_2_1_2_1_
Unit-Cell parameters (Å, °)	
a/b/c	a = 37.3050, b = 54.4190, c = 65.3870
α/β/γ	α = 90.00, β = 90.00, γ = 90.00
Resolution range (out shell) (Å)	41.83–1.7 (1.761–1.7)
No. of reflections	17,298 (1585)
No. of unique reflections	15,182 (1456)
Wilson B-factor	16.59
I/δ (I)	24.5 (5.0)
Redundancy	5.27
Completeness (out shell) (%)	99.82 (98.44)
R-work	0.1691 (0.2558)
R-free	0.1972 (0.2606)

## Data Availability

Atomic coordinates for the reported crystal structure have been deposited with the Protein Data bank under accession number 7YF1.
